# Novel CRISPR–Cas Systems: An Updated Review of the Current Achievements, Applications, and Future Research Perspectives

**DOI:** 10.3390/ijms22073327

**Published:** 2021-03-24

**Authors:** Sweta Nidhi, Uttpal Anand, Patrik Oleksak, Pooja Tripathi, Jonathan A. Lal, George Thomas, Kamil Kuca, Vijay Tripathi

**Affiliations:** 1 Department of Genomics and Bioinformatics, Aix-Marseille University, 13007 Marseille, France; swetanidhi4@gmail.com; 2 Department of Life Sciences and the National Institute for Biotechnology in the Negev, Ben-Gurion University of the Negev, Beer-Sheva 84105, Israel; ushuats@gmail.com; 3 Department of Chemistry, Faculty of Science, University of Hradec Kralove, 50003 Hradec Kralove, Czech Republic; patrik.oleksak@uhk.cz; 4 Department of Computational Biology and Bioinformatics, Jacob Institute of Biotechnology and Bioengineering, Sam Higginbottom University of Agriculture, Technology and Sciences, Prayagraj 211007, Uttar Pradesh, India; pooja.tripathi@shiats.edu.in; 5 Department of Molecular and Cellular Engineering, Jacob Institute of Biotechnology and Bioengineering, Sam Higginbottom University of Agriculture, Technology and Sciences, Prayagraj 211007, Uttar Pradesh, India; jonathanalal@shiats.edu.in (J.A.L.); georgethomas@shiats.edu.in (G.T.)

**Keywords:** CRISPR/Cas9, genome editing, agricultural production, livestock, industrial applications, therapeutics

## Abstract

According to Darwin’s theory, endless evolution leads to a revolution. One such example is the Clustered Regularly Interspaced Palindromic Repeats (CRISPR)–Cas system, an adaptive immunity system in most archaea and many bacteria. Gene editing technology possesses a crucial potential to dramatically impact miscellaneous areas of life, and CRISPR–Cas represents the most suitable strategy. The system has ignited a revolution in the field of genetic engineering. The ease, precision, affordability of this system is akin to a Midas touch for researchers editing genomes. Undoubtedly, the applications of this system are endless. The CRISPR–Cas system is extensively employed in the treatment of infectious and genetic diseases, in metabolic disorders, in curing cancer, in developing sustainable methods for fuel production and chemicals, in improving the quality and quantity of food crops, and thus in catering to global food demands. Future applications of CRISPR–Cas will provide benefits for everyone and will save countless lives. The technology is evolving rapidly; therefore, an overview of continuous improvement is important. In this review, we aim to elucidate the current state of the CRISPR–Cas revolution in a tailor-made format from its discovery to exciting breakthroughs at the application level and further upcoming trends related to opportunities and challenges including ethical concerns.

## 1. Introduction

The very beginning of this exciting Clustered Regularly Interspaced Palindromic Repeats (CRISPR) story dates back to the observations published by a Japanese research group in 1987 [1]. However, Ishino and his colleagues could not explain much about the biological significance of their identified sequences that contained five homologous sequences of 29 nucleotides separated by spacers of 32 nucleotides. The discovery of similar mysterious arrays of regularly spaced repeated sequences was continued by later research groups that gradually revealed their biological significance [2]. The universally accepted CRISPR acronym, Clustered Regularly Interspaced Palindromic Repeats, was coined by a Spanish microbiologist, Mojica [3]. The inquisitive journey that led to the CRISPR discovery, the contributors involved, and their achievements are adequately demonstrated in the literature.

In the journey from the initial observations to the current breakthrough of CRISPR science and technology that has flourished, bloomed, and continued to bear fruits through the past three decades, with many success stories, the scientific attention has gradually turned to reap the benefits of this gene-editing technology while the science that supported this technology is left aside. At this juncture, CRISPR researchers should remember that the foundation of this groundbreaking technology boom was systematic understanding of CRISPR biology and that the abundance of scientific ignorance once left out from this virgin area of molecular biology consists of many gold mines worthy of future research. Hence, along with ongoing advancements in the utilization of CRISPR technology, there is a pressing need to continue exploring its structural features; however, the existing knowledge on these aspects is spread over various articles in the literature. In view of reorganizing the existing information spread and of the pressing need for their systematic analyses, the current review was constructed. This article encourages upcoming CRISPR scientists and subsequently elaborates on the strengths of further scientific inquiry instead of the former scientific ignorance of this novel and unexploited area.

The CRISPR–Cas system is an adaptive immune system in prokaryotes that prevents phage infection by storing memory in the form of viral DNA in bacterial host chromosomes. The system contains viral DNA surrounded by repetitive nucleotide sequences called direct repeats. These direct repeats are surrounded at the near end by sequences encoding proteins called Cas proteins. This system was artificially manipulated in guiding reprogrammed endonucleases to the target gene. CRISPR is one genome editing techniques that modify internal DNA/RNA in a sequence-specific manner and is reprogrammable; CRISPR-associated endonuclease Cas proteins have been used in various ways to precisely modify genes, called gene editing. It has been applied successfully in the field of agriculture, in therapeutics and infectious agents, in food industries, and bioenergy.

The topics covered in the current review focus on (i) elucidating the mechanism of action in different CRISPR systems; (ii) describing the structure of effector complexes in CRISPR; (iii) detailing and summarizing the current benefits of CRISPR–Cas application in plant biotechnology, therapeutics, and the food industry; and (iv) discussing the effects and limitations of the CRISPR technology upon reckless use. In addition, we shed some light on the limitations of CRISPR, providing ethical concerns.

## 2. The CRISPR–Cas System

### 2.1. History of the CRISPR–Cas System

(a) Identification phase: 1987–1993

The first encounter with the five direct repeats containing 24 nucleotide repetitive sequences of the CRISPR–Cas system was in 1987 during the identification of the gene responsible for the conversion of alkaline phosphatase isozyme in *Escherichia coli* [1]. The second occurrence was in *Haloferax mediterranei* containing 30–34 nucleotide sequences, with direct repeats spaced by 35 bp long sequences called a spacer. The direct repeats also contained short, inverted repeats similar to that in the former sequence [4]. During the time phase from 1987 to 1993, the variable sequences in between the direct repeats or spacers of the CRISPR–Cas system intrigued scientists.

(b) Structural and functional characterization phase: 1993–2011

The discovery of similar mysterious arrays of regularly spaced repeated sequences was continued by later research groups in archaea and bacteria and gradually led to their biological significance [2]. During the period from 1993 to 2005, Ruud Jansen and colleagues designated the repeated DNA sequences as Clustered Regularly Interspaced Short Palindromic Repeats (CRISPRs) in 2002, located in the genome near the “DNA repair system” [5]. These nearby genes, earlier understood as part of the DNA repair system, were found mainly accompanying CRISPR genes and, therefore, were termed as CRISPR-associated (Cas) genes. (b) In 2005, two independent research studies linked the spacer’s origin with bacteriophages [6,7]. (c) In 2006, Eugene V. Koonin and his collaborators computationally analyzed the link between the functions of CRISPR and Cas genes as a system and observed a similarity between this system and a prokaryotic RNA interference immune system [8]. (d) The CRISPR–Cas System (CCS) provides resistance to viruses, i.e., after a virus attack [9]. The spacers are stored in the host genome from the phage genomic sequences [2]. In 2011, Kira S. Makarova and coworkers gave an updated analysis of the evolutionary relationships between CRISPR and Cas proteins [10] followed by several papers in this direction.

(c) Application phase: 2011–ongoing

**Figure 1:**
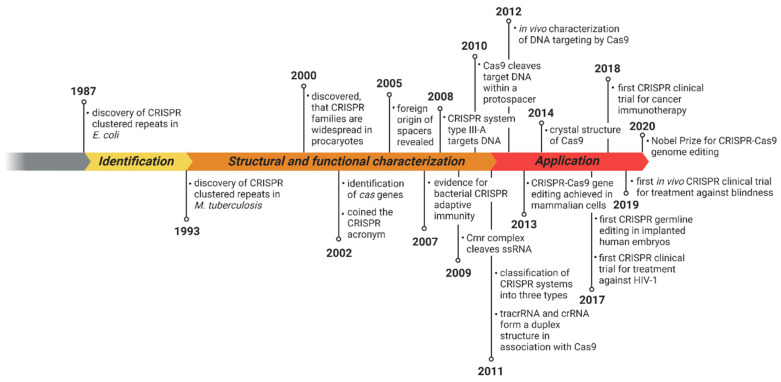
Timeline of the Clustered Regularly Interspaced Palindromic Repeats (CRISPR)–Cas system with important milestones; figure created with BioRender.com (accessed on 15 February 2021).

Thereafter, CRISPR research continued to bloom year after year in thousands of laboratories across the world; the success stories of this novel technology are schematically depicted in few articles. Identification of the CRISPR array and its associates in archaeal and bacterial genomes as efficient defense mechanisms for survival and protection against viral invasion was repurposed as a gene-editing technology to modify eukaryotic genomes that created enormous applications in various field of biology extending from medicine to agriculture. Transfer and reprogramming of the CRISPR–Cas system were conducted immensely in this period. A study showed CRISPR–Cas9 system transfer from *Streptococcus thermophilus* to *Escherichia coli* providing immunity against plasmid and phage infestation [11]. David Bikard and coworkers engineered Cas9 as a transcriptional repressor preventing binding of RNA polymerase (RNAP) to promoter sequences or hindering RNAP [12]. This was proven an asset in the field of gene regulation and synthetic biology and biotechnological applications. With more research, the toolbox of the CRISPR–Cas system improved, and simultaneously, its application flourished. This review focuses on comprehensive detailing of the CRISPR–Cas system’s application in various fields of agriculture, animal husbandry, health, and diseases, etc. The history of the CRISPR–Cas system is summarized in Figure 1. 

### 2.2. Function of the CRISPR–Cas System

In the beginning, the function of CCS remained hidden. The wide presence of CCS in Bacteria and Archaea only informed us about the link between CCS and bacteriophage resistance. The scientific community was therefore more focused on finding the reason behind the former link. Their hard work paid off when the group led by Rodolphe Barrangou and coauthors [9] found that, after a viral infection, new spacer sequences from the invading bacteriophage (genomic inserted viruses) were inserted into the genome of the host. Any addition or removal of these inserted spacer sequences altered the phenotype of phage-resistance of the cell [9]. Many influential works led us to deduce the path of the functions. The important functional steps of the CRISPR system are adaptation, expression, and interference.

**Figure 2:**
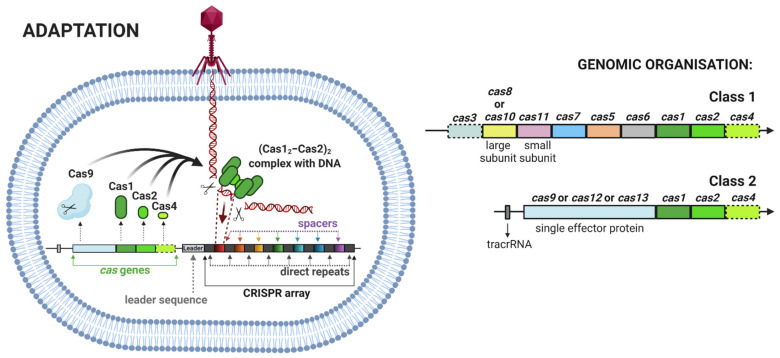
**Left side:** CRISPR adaptation step [2,9,10,14]. **Right side:** Generalized genomic organization in class 1 and class 2 systems. A dashed outline indicates that the gene is dispensable or missing in some subtypes or variants [14]. The figure was created with BioRender.com (accessed on 20 March 2021).

**Figure 3:**
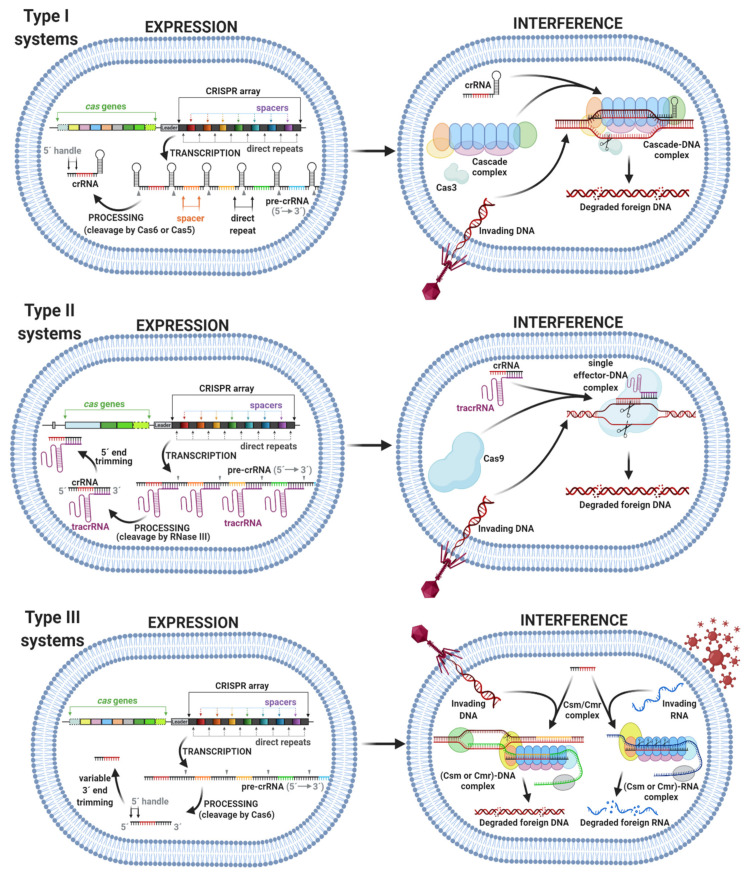
CRISPR expression and interference step in type I (top), type II (middle), and type III (bottom) systems. The figure was created with BioRender.com (accessed on 15 February 2021).

(1) Adaptation, also termed as insertion or acquisition, is a process of foreign DNA sequence incorporation into CRISPR arrays (Figure 2). Integration of a new spacer is mediated by the heterohexameric protein complex ((Cas1_2_–Cas2)_2_) to the leader sequence of the CRISPR array. There are two different types of spacer acquisition system named type I and type II. A type I system utilizes integration host factor (IHF) bonded to the leader sequence, which induces DNA bending. This bending enables the (Cas1_2_–Cas2)_2_ complex to perform an initial cleavage for insertion of the spacer. In a type II system, the leader anchoring sequence (LAS) of the leader is recognized using the Cas1 protein of the (Cas1_2_–Cas2)_2_ complex and, then, the polar spacer is inserted [13].
(2) Expression, also termed CRISPR RNA (crRNA) biogenesis or crRNA processing, represents the transcription of a CRISPR array into a long precursor CRISPR RNA (pre-crRNA). Further processing involves the cleavage of pre-crRNA within each direct repeat sequence to afford shorter, mature CRISPR RNAs (crRNAs). Some crRNAs can further undergo 5′ end or 3′ end trimming. The enzymes involved in crRNA processing differ among types, in some cases, even among subtypes of the CRISPR–Cas system (Figure 3) [15].
(3) Interference involves the formation of a multiprotein effector complex or single effector protein. The multiprotein effector complex is typical for class 1 systems and consists of multiple Cas proteins and crRNA. The single effector protein is utilized in class 2 systems and contains only a single Cas protein with crRNA. The primary purpose of both complexes is to recognize the same or very similar sequences in the genome of the invading virus or plasmid. After recognition, the invading genome is cleaved by the complex and inactivated [14]. The interference step of some systems requires recognition of a protospacer adjacent motif (PAM) in the invading genome. The PAM is a short DNA sequence that is not present in the bacterial host genome. Hence, PAM is an essential targeting sequence to bind for some Cas proteins, followed by cleavage (Figure 3) [16].

### 2.3. Structure of CRISPR Locus and Classification of Cas Proteins

The chromosomes of prokaryotic species generally contain one CRISPR locus [3]; however, several species contain more (up to eight) CRISPR loci [17]. The CRISPR locus consists of three major parts:

- The CRISPR array consists of short, direct repeats bordered with spacers. The direct repeats are nucleotide sequences in the genome with identical sequence and length. The sequences of direct repeats can be similar in related species, but overall diversity among the species is wide. The average size of the repeats is 32 bp; however, the size may vary from 21 to 47 bp. The spacers are nucleotide sequences with a fixed length, but they are highly variable in sequences. The average size of spacers is from 20 to 72 bp [18].
- The leader sequence is commonly adjacent to the CRISPR array and is involved in adaptation and transcription. These regions exhibit only limited conservation in sequence. It was observed that leaderless CRISPR loci are inactive in adaptation but still able to contribute to crRNA-directed interference [19].
- *CRISPR-associated* (*cas*) genes represent a cluster of genes in varying orientation and order that code corresponding Cas proteins (Table 1). In summary, 93 different *cas* genes have been identified until now. These genes were classified into 35 families based on the sequence similarities [20]. Cas proteins play a major role in the acquisition and destruction of foreign sequences (Table 2).

### 2.4. Classification of the CRISPR–Cas System

Classification of the CRISPR–Cas system is essential for understanding the origin and further research on the CRISPR system. The classification is based on differences in Cas protein composition and sequence divergence between the effector complexes. The evolutionary classifications of the CRISPR–Cas system were reported by Makarova et al. in 2011 [10], in 2015 [20], and most recently in 2020 [14]. These reports demonstrate an extensive interest in this area. According to the classification reported in 2020 [14], the CRISPR–Cas system is divided into 2 classes, 6 types, 33 subtypes, and several variants.

The class 1 system consists of type I, type III, and type IV systems (Table 3). This class utilizes an effector complex composed of multiple Cas proteins and crRNA in the interference step. The type I system contains 7 subtypes (I-A, I-B, I-C, I-D, I-E, I-F, and I-G) including several variants. The pre-crRNA in type I systems contains palindromic repeats that are either unstructured (subtypes I-A and I-B) or form hairpin structures (subtypes I-C, I-D, I-E, and I-F). The cleavage of pre-crRNA in a type I system is generally mediated by Cas6 protein; however, cleavage in subtype I-C systems is mediated by a Cas5 protein. The CRISPR-associated complex for an antiviral defense (Cascade) complex is an effector complex for the interference step in type I systems. The Cascade complex usually contains Cas3, Cas5, Cas7, Cas8, and other Cas proteins in different combinations according to subtypes. A key component of the Cascade complex in type I systems, responsible for foreign DNA cleavage, is Cas3 protein. In some subtypes of type I systems, Cas3 is fused with Cas2 protein [14,22]. The type III system consists of 6 subtypes (III-A, III-B, III-C, III-D, III-E, and III-F); some of them include reverse transcriptase in the adaptation module. The cleavage of pre-crRNA in type III systems is mediated by Cas6 protein; however, most of the subtypes lack the *cas6* gene and use the Cas6 protein provided *in trans* by other CRISPR-*cas* loci. The effector complex in subtypes III-A, III-D, III-E, and III-F is a Csm complex composed of Csm/Cas proteins and crRNA. Subtypes III-B and III-C comprise the Cmr complex composed of Cmr/Cas proteins and crRNA. Effector complexes of subtypes III-A, III-B, and III-C cleavage foreign RNA/DNA and, in subtypes III-D and III-E, are predicted to cleavage RNA, whereas in subtype III-F, effector complexes are predicted to cleavage DNA [14,20,22]. A type IV system contains 3 subtypes (IV-A, IV-B, and IV-C). These systems usually lack Cas proteins in the adaptation step and target cleavage of the foreign genomes. Processing of pre-crRNA is usually mediated by a unique Cas6 protein. It has been proposed that effector complexes of type IV systems contain Cas5, Cas7, and large subunit (Csf1) proteins. Recent studies suggest that type IV CRISPR–Cas systems could be highly diverged derivatives of types I or III systems [14,23].

Class 2 system consists of type II, type V, and type VI systems (Table 4). In contrast to class 1, the effector complex of class 2 systems is a single, large, multidomain Cas protein bonded with crRNA. Type II system involves 3 subtypes (II-A, II-B, and II-C). Processing of pre-crRNA in type II systems is mediated by the coordinated action of three factors: RNase III (non-Cas protein), trans-activating CRISPR RNA (tracrRNA), and Cas9 protein. Alternative processing of pre-crRNA was discovered in subtype II-C systems. The single effector protein in type II systems is Cas9 with two nuclease domains responsible for cleavage of one strand of the target DNA [14,22]. A type V system contains 10 subtypes (V-A, V-B, V-C, V-D, V-E, V-F, V-G, V-H, V-I, and V-K), with the Cas12 protein as a single effector complex. Processing of pre-crRNA in subtype V-A is performed by the effector complex, whereas in several type V subtypes, the processing is mediated by RNase III. Both strands of target DNA are cleaved by one domain of Cas12 protein. A type VI system involves 4 subtypes (VI-A, VI-B, VI-C, and VI-D). Single effector complexes in type VI systems are Cas13 proteins that differ from the other effector complexes in class 2 systems. The processing of pre-crRNA is performed by the effector complex. All effector complexes contain two higher eucaryotes and procaryote nucleotide-binding (HEPN) domains, which provide RNase activity. The HEPN domains of the Cas13 protein in type VI systems cleavage foreign RNA [14].

### 2.5. Structure and Function of Effector Complexes

#### 2.5.1. Effector Complexes of Class 1

**Figure 4:**
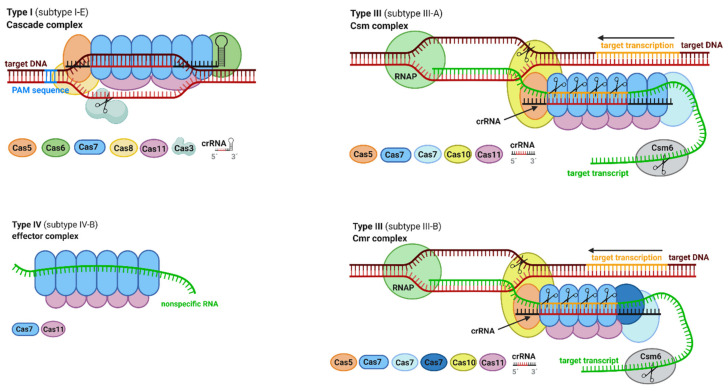
Selected multiprotein effector complexes of CRISPR class 1 systems. Figure was created with BioRender.com (accessed on 15 February 2021).

The Cascade complex is a multiprotein complex for interference in type I systems (Figure 4). Among all subtypes in type I systems, subtype I-E of *Escherichia coli* is the most thoroughly characterized. Moreover, subtype I-E contains a full complement of subunits that are present in another type I systems; therefore, it is a unique model of the Cascade complex. The formula of the Cascade type I-E CRISPR–Cas system can be described as follows: (Cas5e)_1_–(Cas6e)_1_–(Cas7e)_6_–(Cas8e)_1_–(Cas11e)_2_ with a molecular weight of 450 kDa. The structure of the Cascade I-E type consists of 6 Cas7e proteins, which form a helical backbone with integrated crRNA that is capped with the Cas5e protein. Furthermore, two Cas11e proteins termed “small subunits” interact with the Cas7e backbone. The protein Cas8e also termed a “large subunit”, interacts with the Cas5e, Cas6e, and Cas7e proteins and forms the tail of the Cascade complex. The Cas8e protein recognizes the PAM sequence in the target DNA and is responsible for the initial local unwinding of target DNA. Further conformational changes induce the participation of Cas3 nuclease for the final cleavage of the target DNA. Although the general composition and Cas3 nuclease involvement among the Cascade complexes of type I systems are similar, several subtypes manifest significant differences. Notably, subtype I-C expresses a minimal architecture of the Cascade complex. The backbone in the subtype I-F Cascade complex contains an unusual helical pitch [20,24].

Csm (in subtype III-A) and Cmr (in subtype III-B) complexes are multiprotein interference complexes similar in structure and protein composition to the Cascade complex of type I systems (Figure 4). These complexes are best characterized among the type III systems. In contrast to the Cascade complex, the Csm and Cmr complexes cleave both invading RNA and DNA. The backbones of Csm and Cmr complex structures consist of Cas7-family proteins fused with crRNA, which is capped with the Cas5 protein. Cas11 represents a small subunit, while the large subunit is a Cas10 protein. Cleavage of the target genome begins by binding the type III effector complex to a nascent target transcript in a crRNA-dependent manner. The single-stranded RNA (ssRNA) is cleaved at every sixth nucleotide by Cas7 subunits, whereas DNA cleavage is mediated by Cas10 protein and strictly requires transcription of the target in both systems [10,24].

The first effector complex of type IV systems was described in 2020 by Zhou et al. in subtype IV-B. The structure of the subtype IV-B CRISPR effector complex involves a helical backbone formed by 7 Cas7-like proteins, bundled with 5 Cas11 proteins (Figure 4). Interestingly, the Cas7 protein of subtype IV-B exhibits significant similarities to Cas7 of the Csm complex in subtype III-A. The RNase activity of the Cas7 protein in subtype III-A is also proposed for Cas7 of subtypes IV-B. In addition, the structure of the Csm complex with crRNA strongly resembles the structure of the IV-B effector complex with nonspecific RNA. The ability of the subtype IV-B CRISPR effector complex to bind nonspecific RNA remains unclear. Moreover, in contrast to the Csm complex, one of the catalytic residues (Asp42) of the Cas7 subtype IV-B complex is in a noncompatible position for target RNA cleavage. The study reported by Zhou et al. reveals that type IV systems evolved from ancestors of type III-like systems [25].

#### 2.5.2. Effector Complexes of Class 2

**Figure 5:**
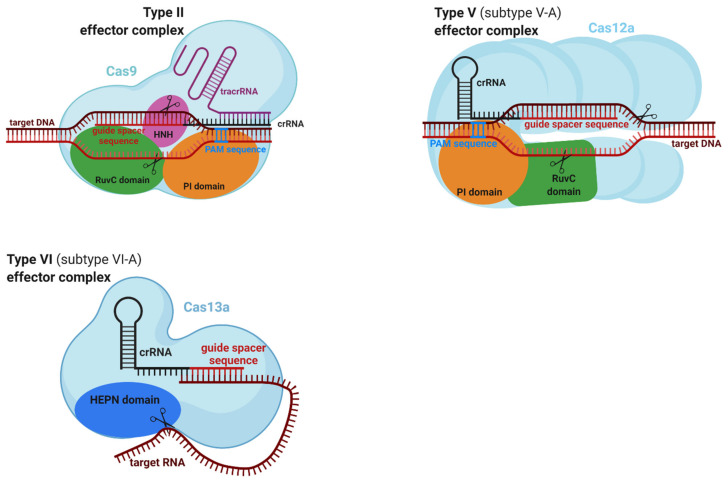
Selected single effector complexes of class 2. The figure was created with BioRender.com, accessed on 15 February 2021.

The CRISPR–Cas9 system is utilized in the type II system and provides its function via a single effector complex comprising a Cas9 protein, crRNA, and tracrRNA (Figure 5). The target DNA interacts with two different areas of the effector complex. One bond is mediated by a guide spacer sequence (18–24 nt) of crRNA with a target DNA complementary sequence. The PAM interacting (PI) domain of the effector complex binds to its recognition site upstream of the PAM sequence. Double-stranded breaks of the target DNA perform two domains of the complex. The HNH (histidine–asparagine–histidine) nuclease domain cleaves the target strand, whereas the RuvC nuclease domain cleaves the nontarget strand of foreign DNA [26].

The CRISPR–Cas12a system in subtype V-A systems performs its functions via a single effector complex, which is composed of the Cas12a protein and crRNA (Figure 5). Two areas of the effector complex interact with target DNA. The PI domain of the effector complex binds to its recognition site downstream of the PAM sequence. The second interaction is between the guide space sequence (23–25 nt) of the crRNA and a complementary sequence of the target DNA. Double-stranded breaks in invading DNA are mediated by two domains. The Nuc domain cleaves the target strand and the RuvC domain nontarget strand of target DNA [26].

The CRISPR–Cas13a system is utilized in subtypes VI-A, and its function is provided via a single effector complex, which contains Cas13a protein and crRNA (Figure 5). The guide spacer sequence (22–30 nt) of crRNA recognizes complementary sequences in foreign RNA. The CRISPR–Cas13a system does not require a PAM sequence, but some subtypes require a single base-specific protospacer flanking site (PFS) sequence. However, there are also subtypes of CRISPR–Cas13 systems that do not require specific PFSs. In contrast to Cas9 or Cas12, there are no HNH and RuvC domains in the Cas13 protein. Therefore, DNA is not a target molecule of Cas13. The cleavage of target RNA is mediated by the HEPN domains of the complex [26].

## 3. Applications of CRISPR–Cas Systems

The development of the RNA-programmable site-specific CRISPR–Cas9 system in gene editing methods published in the *Science* journal [27] inspired countless potential applications and won many internationally acclaimed awards including a Nobel Prize. The Royal Swedish Academy of Science decided to award the Nobel Prize in Chemistry 2020 to Emmanuelle Charpentier and Jennifer A. Doudna “for the development of a method for genome editing”.

**Figure 6:**
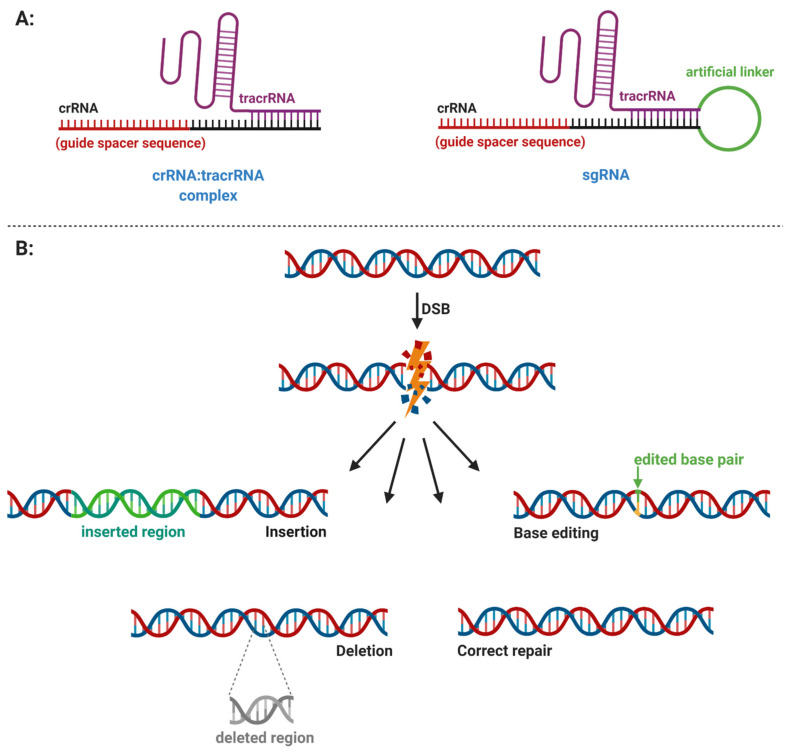
(**A**) General structure of the CRISPR RNA (crRNA)–trans-activating CRISPR RNA (tracrRNA) complex and artificial single-guide RNA (sgRNA). (**B**) CRISPR–Cas-assisted gene editing. The figure was created with BioRender.com (accessed on 15 February 2021).

Both classes of CRISPR systems have a significant potential for genome editing; however effector complexes of class 2 systems are more simplified and, thus, they are preferable in genetic engineering. Genome editing via CRISPR class 2 systems utilizes an artificial single-guide RNA (sgRNA) that consists of tracrRNA, crRNA, and an artificial RNA linker [21]. In contrast to artificial sgRNAs, a natural guide RNA (gRNA) in type II systems, also termed a crRNA–tracrRNA complex consists only of tracrRNA and crRNA without the linker (Figure 6A). In addition, a modified sgRNA, which carries both sequences—one to generate double-stranded breaks and the second for a homology-directed repair—is termed a chimeric single-guide RNA (cgRNA). Further modifications were carried out on the Cas9 active sites. These modifications in the CRISPR–Cas9 system improved some mechanisms compared to the wild-type system, e.g., in DNA targeting or the introduction of single-stranded breaks (SSB) in DNA instead of double-stranded breaks (DSB) [21].

The CRISPR-mediated genome editing introduces DBSs close to the PAM sequence (Figure 6B). DBSs in eukaryotes are repaired by non-homologous end joining (NHEJ) or homology-directed repair (HDR) pathways. The NHEJ is an error-prone repair mechanism in which insertions and deletions (indels) occur at the DSB junctions. The HDR pathway requires the presence of a homologous DNA template but repairs DSB with high precision. HDR allows for inserting novel genes (knock-in) or “knockout” existing genes. The homologous sequences can be provided exogenously and utilized to target genome editing [21,28].

Base editing belongs to genome editing methods, which can generate precise point mutations in DNA or RNA without generating DSBs. The base editing method requires a DNA donor template or relies on HDR. The DNA base editors are divided into two classes: cytosine base editors (CBEs) and adenine base editors (ABEs). The CBEs convert a C–G base pair into a T–A pair, whereas ABEs convert the A–T pair into a G–C base pair. Both base editors thus can provide all four possible conversions (C→T, A→G, T→C, and G→A) [21,29].

### 3.1. In Plant Biotechnology

According to a report published in the Global Hunger Index, 2019, the increasing climate change impacts food systems adversely, weather-related disasters reduce crop yield, and excess carbon dioxide generation decreases the nutritional value of crops. To combat food scarcity, agricultural productions must be enhanced by the combination of traditional plant breeding (whole-genome editing) and innovative techniques such as molecular plant breeding (targeted genome editing) and specific gene editing. Targeted genome editing has increased productivity: increasing the grain size, weight, number, protein content, tiller spread, and tiller number in rice and wheat [30,31,32] and quality of crops in rice [30,31] and corn [33]. Modified crops using the CRISPR–Cas system were targeted to reduce the levels of toxic steroidal glycoalkaloids, enhancing the color and shelf-life of fruits and vegetables and making them commercially attractive. The modifications further involved an increase in amylose, starch, good fats such as oleic acid levels; fragrance improvements; a decrease in gluten proteins and unsaturated fatty acids content; etc. [34,35,36,37].

#### 3.1.1. Resistivity to Stress

Stress significantly reduces the productivity of agricultural crops. Stress in plants can be divided into two categories: abiotic stress is caused by different factors including drought, floods, temperature extremes, salinity, heavy metals, radiation, etc. In contrast, biotic stress involves attacks by various pathogens, e.g., viruses, bacteria, fungi, herbivores, and others. Crops such as rice, tomato, cucumber, grapefruits, etc. have been modified by inducing mutation to protect from abiotic [38,39] and biotic stresses [40]. The site-specific genomic mutation was performed earlier by DNA-binding endonucleases such as zinc finger nuclease (ZFN) and transcription activator-like effector nucleases (TALEN) but has limitations [41]. For the first time, the CRISPR–Cas system was used for genome editing in rice (*Oryza sativa*), wheat (*Triticum aestivum*), *Nicotiana benthamiana,* and *Arabidopsis thaliana* [42].

A customized sgRNA-Cas9 system has been widely used by Shan et al. in genome modification in rice (*Oryza sativa*) and wheat (*Triticum aestivum*); the first plants showed the ease of genome editing [38]. Cas12a (formerly termed Cpf1) is advantageous over Cas9 in plant genome editing because sgRNA–Cas12a requires shorter guiding nucleotides, creates larger deletions at the target sites, and helps in NHEJ mediated donor DNA insertion [43].

Before genome editing, CRISPR–Cas reagents, i.e., DNA, sgRNA, and Cas proteins, must be delivered to the plants. The delivery is performed by protoplast transfection or *Agrobacterium*-mediated or biolistic transformations. *Agrobacterium tumefaciens*-assisted sgRNA–Cas9 targeting of plant herbicide resistance gene *BEL* (*Bentazon Sensitive Lethal*) in rice has been demonstrated to obtain transgenic plants sensitive to bentazon, a herbicide with a mutagenesis efficiency of 2–16% [44]. Geminiviruses also serve as a good vector for transfer and expression of the sgRNA–Cas9 construct [45]. Xiang Ji and collaborators mutated genomic sequences and restricted viral load in *Nicotiana benthamiana* using beet severe curly top virus (BSCTV), one Geminivirus [45]. The sequence-specific interference of Geminiviruses by CRISPR–Cas tools boosting the immunity of the plants has been reported [46]. This virus-based editing, termed virus genome editing (VIGE), has been used to target the genome and to create mutations but fails to transmit these mutations to the next generations [45]. However, in *Arabidopsis*, Zhengyan Fenga and coworkers demonstrated the mutation and heritability of five endogenous target genes—*brassinosteroid insensitive 1* (*bri1*), *jasmonate-zim-domain protein 1* (*jaz1*), *gibberellic acid insensitive* (*gai*), *magnesium chelatase subunit i* (*chli*), and *transparent testa 4* (*tt4*)—in addition to the *apetala1* (*ap1*) gene [47]. Similarly, the CRISPR–Cas tools can be used for regulating the genes responsible for the epigenetic modification, methylation, and/or demethylation, inducing and repressing the genes simultaneously [48].

CRISPR–Cas13a efficiently targets RNA viruses, mostly plant viruses. Aman et al. [49] utilized LshCas13a (a Cas13a variant from *Leptotrichia shahii*) for targeting Turnip mosaic virus (TuMV), a *Potyvirus*, in *Nicotiana benthamiana*, an *Agrobacterium* containing green fluorescent protein (GFP) expressing TuMV with LshCas13a tagged with C-terminal nuclear localization sequence (NLS) and various crRNAs targeting different parts on the viral genomes. After seven days of infiltration, two out of the four crRNA showed a 50% reduction in GFP expression. Cas13a was also used for precise RNA modifications and visualization. Dead Cas13a was formed by a point mutation at the HEPN domain responsible for RNA targeting. Abudayyeh et al. [50] used dLwaCas13a (a dead Cas13a variant from *Leptotrichia wadei*) fused with fluorescent proteins to visualize specific transcripts in live cells.

Hybrid breeding is another method that increases crop productivity including improvements in hybrid wheat seed production. Indeed, hybrid crops used today are effective high-yield varieties; however, the production of hybrid seeds requires sterilization to prevent self-pollination. Similarly, for precision plant breeding, knockout, a process of replacing the undesired gene with the desired gene, is performed [42]. CRISPR–Cas has been employed to produce thermosensitive male-sterile *TMS5* lines in rice [51] and maize [52], *Ms45* in wheat, etc. [53]. The male-sterile lines produced a high-quality hybrid variety. Similarly, haploid rice has been obtained by knockout of the *OsMATL* gene [54]. Furthermore, the CBE has been used to confer herbicide resistance in rice [55], *Arabidopsis* [56], and watermelon [57]. Further CRISPR–Cas genome editing studies in plants are summarized in Table 5.

#### 3.1.2. Prospects in Plant Gene Editing

CRISPR–Cas is a simple, precise, and user-friendly toolbox for plant gene editing. The tools can be explored in various ways in the future: (i) The genome structure and gene function can be elucidated in plants including visualizing gene loci in humans. (ii) In synthetic biology, CRISPR can be used for introducing desired foreign genes with sgRNA with specific strong promoters and transcriptional regulators in the plant for novel products and functions. In the C4 rice project [111], where changes in the photosynthetic apparatus failed to increase carbon fixation, CRISPR can be useful in editing the photosynthetic apparatus and in fixing the carbon efficiently [112]. (iii) Easy multiple gene-editing simultaneously at multiple loci in domesticated and non-domesticated plants is the need of the hour to provide global food security. Increasing the speed of domestication of crops can prevent loss of diversity in plants and can feed an increasing population such as in the case of domestication of wild tomato [113]. (iv) The specificity and efficiency of gene editing can be improved by homology-directed repair [114]. (v) Weeds and pests can be eliminated with the help of gene drives. **(vi)** Regulatory authorities and researcher’s societies should work in coordination and should not allow random, illicit genome editing and gene drives until a strong controlled framework is available. The above all-important applications demonstrate the wide use of the CRISPR tool and all the improved edited plants produced are only the tip of the iceberg. Hence, genome editing by CRISPR–Cas9/Cas12a and plant breeding will help society overcome food scarcity for exponentially increasing populations [48]. Conclusively, CCS can tailor plants for survival in unfavorable conditions, can provide ample food for all, and can make the world a good place to live.

The CRISPR–Cas13a system is used to engineer resistance against plant RNA viruses. Multiple genes are targeted by this system using multiple guide RNAs and by expressing them under polymerase II, thus enhancing targeting efficiency. Due to high mismatch, high sensitivity of this system, differentiation, and proper intervention is possible between highly similar viral strains. This system can also detect a single copy of RNA with high specificity and sensitivity, a requirement for viral detection in the early detection of the virus [115]. Cas13a along with CRISPR–Cas9 was employed in targeting the RNA and DNA viruses directly in non-transgenic cucumber, providing plant dual immunity [68].

### 3.2. In Therapeutics

#### 3.2.1. For Treating Genetic Diseases

The CRISPR–Cas system has been widely used in the correction of human genetic diseases including Duchenne muscular dystrophy (DMD) [116], α-1 antitrypsin deficiency (AATD) [117], hemophilia [118], hematopoietic diseases [119], and hearing loss [120]. The genetic corrections are carried out by CRISPR–Cas9-based hematopoietic stem and progenitor cells (HSPCs) [121], recovering pathogenic mutation in induced pluripotent stem cells (iPSCs) in normal hemoglobin, etc. [122]. DMD is caused by a mutation of the dystrophin gene. In DMD patients, the exon 50 deletion coding rod domain of dystrophin places exon 51 and the preceding exons out of frame. In addition, the deletional mutation of exon 44 places the dystrophin genes out of frame. The *in vivo* delivery of Cas9 and sgRNA was performed with adeno-associated virus serotype 9 (AAV9). Thus, the group using AAV9-mediated Cas9 and sgRNA skipped or restored the exon 50 deletions and prevented mutations of exon 44 using CRISPR-mediated skipping of the surrounding genes [116]. AATD is a hereditary liver disorder caused by a mutation in the gene encoding the serine protease inhibitor (*SERPINA1*). Severe patients homozygous for this deficiency have a lump of proteins in the liver and reduced circulation of α-1 antitrypsin. In a mouse model of AATD, researchers destroyed the mutant version of the *SERPINA1* and obtained dissolution of liver fibroids and mutant protein aggregation [117].

Blood disorders, such as β-thalassemia and sickle cell diseases (SCD), are caused by the structural or reduced production of β-chains, thus decreasing the oxygen-carrying capacities of hemoglobin. Bone marrow cells are harvested using CRISPR–Cas technology for the production of fetal hemoglobin to fight against the symptoms of such diseases. The 200 bp *BCL11A* erythroid enhancer including GATAA motif deletion leads to increased production of γ-hemoglobin expression in K562 cells [119]. Fetal hemoglobin with γ chain is a natural form of the oxygen-carrying protein that binds oxygen better than adult hemoglobin. Hearing loss due to mutant *Tmc1* was targeted by *Staphylococcus aureus* Cas9 in *Beethoven* mice and a DFNA36 human cell line. *Tmc1* is a gene encoding a pore-forming subunit of mechanosensory transduction channels in inner-ear hair cells. The AAV-mediated delivery of Cas9 prevented deafness in *Beethoven* mice up to one-year post injection [120].

#### 3.2.2. For Management of Infectious Diseases

Viruses are known to cause latent infections, which include human immunodeficiency virus (HIV), herpes simplex virus (HSV), Epstein–Barr virus (EBV), human cytomegalovirus (HCMV), Kaposi’s sarcoma-associated herpesvirus (KSHV), John Cunningham virus (JCV), etc. [123]. For many infectious viral diseases such as respiratory syncytial virus (RSV), influenza, and EBV, there are no effective vaccines that clear the viral genome completely from the host. The CRISPR–Cas system represents a promising tool to fight viral infections. It is also predicted that CRISPR can edit the human genome to prevent people from being infected.

##### Control of Human Immunodeficiency Virus (HIV)

Human immunodeficiency virus DNA is reversely transcribed and depends upon a host for its replication similar to that in any other RNA virus [124] and integrates into the genome acting as a latent reservoir, which creates a problem in its eradication [125]. Highly active antiretroviral therapy (HAART) is the current method used to stop replication but fails to fight the latent infection. Therefore, for fighting latent infections due to HIV, CRISPR–Cas technology has been widely prevalent in destroying HIV proviruses [125].

Targeting of the various viral gene can be summarized in few points: (i) In a study, long terminal repeat (LTR) regions on both ends of viral genes were targeted by CRISPR–Cas9 into HIV-1 LTR expression-dormant and inducible T cells. The results showed a significant loss of LTR expression due to cleavage and mutation of LTR target sites [126]. (ii) The glycoprotein CD4 on T cells and CC chemokine receptor type 5 (CCR5; formerly termed CKR5) or CXC chemokine receptor type 4 (CXCR4) facilitate HIV-1 entry into the host cell, and therefore, HIV-1–CCR5 interactions can check the entry of HIV and treatment of AIDS. Hence, in a study, the Cas9 was reprogrammed to destroy plasmid-encoded human CCR5 and obtained 33% mutation at the *CCR5* locus [127]. (iii) CXCR4 binds to the gp120 envelope protein and mediates viral infection in the CD4^+^ T cells. Therefore, a study found that targeting two sites in *CXCR4* led to ablation of CXCR4, making the modified cells resistant to X4-type HIV-1 infection [128]. (iv) Simultaneous genome editing of *CXCR4* and *CCR5* by CRISPR–Cas9 in various T cell lines and primary CD4^+^ T cells showed no off-target effect and cytotoxic effects on cell viability [129,130]. Dash and coworkers targeted the HIV-1 subgenomic particles surrounding the LTR and *gag* gene and reported the removal of proviral DNA without any off-target effects [131]. A study by He Jiankui showed that deleting 32 amino acids coding CCR5 and known for providing resistance to HIV-1 infection does not protect from all strains of HIV [132]. Although, He reported in 2018 the birth of the first genetically edited babies Lulu and Nana, the sequencing of DNA from their placenta, umbilical cord, and cord blood to assess on- and off-target mutations showed off-targeted effects and mosaicism in both babies. Neither of them exhibited *CCR5Δ32* variants protective against HIV. Cas12a completely inactivates HIV with sgRNA and stands as a promising tool for genome editing with high specificity and activity [133].

##### Detection of Severe Acute Respiratory Syndrome Coronavirus 2 (SARS-CoV-2)

Researchers are using different CRISPR–Cas technologies for the early, rapid, and efficient detection of viruses. In a recent study, sequence-specific recognition of HIV-1 was performed by CRISPR–Cas-assisted nanopores [134]. Similarly, the recent outbreak of a novel coronavirus SARS-CoV-2 responsible for the global COVID-19 pandemic is detected by all-in-one dual CRISPR–Cas12a (termed “AIOD-CRISPR”) assay in which a pair of crRNAs was introduced to detect nucleic acids of SARS-CoV-2 (DNA and RNA) and HIV [135]. Currently, the novel coronavirus SARS-CoV-2 deadly outbreak has 2,642,826 confirmed deaths and 119,220,681 confirmed cases worldwide according to WHO data (5:13 pm CET, 14 March 2021) (https://covid19.who.int/ (accessed on 14 March 2021)). The rapid detection of the novel pathogen has laid out technological challenges for the scientific field. Hou et al. were able to develop a highly sensitive CRISPR-based diagnostic tool for the detection of strains genetically such as *SARS*-*CoV*. Although research is in evaluation, it promises shorter run times and higher sensitivity detection than RT-PCR [136].

##### Management of Other Infectious Diseases

Viral diseases are difficult to treat due to the high mutation rate and high latency of infections. One such virus is herpes simplex virus type 1 (HSV-1), which can perform either lytic or latent infections [137]. The virus expresses *latency-associated transcript* (*LAT*) genes during latency and remains protected in the cytoplasm or nucleus without or with fewer symptoms in the host. However, reactivation of the viruses into lytic infection implicates disease and, in some cases, death. Rohem et al. designed a mixture of sgRNA that targets ICP0, ICP4, and ICP27 immediate proteins crucial for viral replication and that eliminates viral infections [137].

MicroRNAs (miRNAs) are noncoding, short RNAs found in Epstein–Barr virus (EBV) that regulate viral and host cell gene expression. The EBV-miRNAs are associated with several biological functions also in the development of cancer [138]. Further study revealed that sgRNA successfully targeted and downregulated EBV miRNAs such as miR-BART5, miR-BART6, and miR-BART16 in latency. For lytic infection, the authors designed sgRNA against the viral EBV nuclear antigen 1 (EBNA1) and several genes under the EBV origin of replication (OriP). A similar approach was tested for HSV-1 and human cytomegalovirus (HCMV) [139]. A decrease in viral replication and abrogation of replication upon reactivation from latency in HSV-1 were observed. The adenoviral-mediated specific Cas9 targeting of gene encoding latency-associated nuclear antigen (LANA) decreased episomal load, which is necessary for the maintenance of Kaposi’s sarcoma-associated herpesvirus (KSHV) in the host [140], and similarly targeted genes responsible for the production and the fitness of HSV-1 and HCMV abrogate viral replication [139].

In a study for HCMV, singleplex (1 cgRNA) and multiplex (3 cgRNA’s) lentivirus-mediated targeting of the start codon and *UL122/123* gene were performed, respectively. The *UL122/123* gene encodes for early proteins responsible for viral replication. In singleplex, site-specific cleavage was induced in the *UL122/123* gene by one cgRNA, whereas in multiplex, 3300 bp were deleted in the *UL122/123* gene using three cgRNAs. This led to a prominent reduction in immediate-early (IE) protein expression. The new virion production was reduced by CRISPR–Cas9 technology up to 98% [141]. CRISPR–Cas9 has been used to replace protective vaccines with endogenously produced antibodies in primary human B cells, resulting in potency and protection against influenza, EBV, and respiratory syncytial virus (RSV) infections [142].

Wollebo and coworkers targeted human polyomavirus (JCV) genes encoding viral early proteins and T antigens. The proteins were responsible for viral reactivations and lytic infections. The analysis revealed that targeting of N-terminal of T antigens and mutations in viral proteins perturbed the replication and function of viral proteins, preventing viral replication in the cells [143]. Conclusively, the specificity and precision of CRISPR–Cas technology and development in the delivery agent of the CRISPR–Cas system in an animal model can cure the herpesvirus infection.

#### 3.2.3. For Management of Cancers

Cancer is a disease characterized by multiple oncogene and epigenetic mutations. CRISPR–Cas technology, a versatile technology for gene editing can be easily transferred to the cells and has extensive application in cancer biology and oncology [144]. The CRISPR–Cas system represents a suitable tool for the exploration of different oncological mechanisms including tumor occurrence, development, and treatment by repairing mutations and knockout genes. The detection of cancer can be conducted by Cas13a using specific high-sensitivity enzymatic reporter unlocking (SHERLOCK). The SHERLOCK platform is a rapid and high-sensitive method to detect nucleic acids. In this, recombinase polymerase amplification of DNA or RT-RNA is performed, followed by T7 RNA polymerase transcription. The method detected these transcribed nucleic acids using Cas13a reporter probes, and fluorescence was measured [145]. The different strategies followed to edit the genome caused mutations that facilitated proto-oncogene inhibition and tumor suppressor gene activation [146,147].

(i) Inhibition of Gain in function mutation (GiF) of proto-oncogenes: CRISPR–Cas-mediated gene knockout of *CD38* in human lung adenocarcinoma A549 cells inhibited anchorage-independent cell growth and development, cell invasion, and xenograft growth in nude mice [148]. One example of GiF is inhibition of the PIK3C3 and FGFR pathways by a first-class compound MPT0L145 that exhibited significant anti-bladder cancer activity via autophagy-dependent cell death. MPT0L145 further exhibited stronger cytotoxicity compared to other PIK3C3 inhibitors. However, CRISPR–Cas9-mediated knockout of the *ATG5* gene reversed MPT0L145-induced cell death by autophagy introduction [149].

(ii) Inhibition of Loss in function mutation (LiF) of tumor suppressor genes: the LiFs of *Acvr1b*, *Acvr2a*, and *Arid2* behaved as a tumor suppressor in colorectal cancer. It was also observed that mutations in the receptors of activin and transforming growth factor-β (TGF-β) promoted the formation of tumors synergistically [150]. BAP1 is a tumor suppressor and regulates chromatin accessibility. The LiF of BAP1 by CRISPR–Cas9 in human cholangiocyte organoids resulted in the acquisition of malignant features upon xenotransplantation. The result demonstrated a key aspect of BAP1’s tumor suppressor function [151]. LiF or GiF CRISPR–Cas9 gene knockout aides in cervical cancer detection, prevention of HPV infection, reduction in occurrence and death due to ovarian cancer, and deeper understanding of endometrial cancer [28].

(iii) Immunotherapy is a biological therapy that strengthens our flawed immune cells to fight cancer. Immune cells, such as T cells, are genetically modified using viral or nonviral vectors to form chimeric antigen receptors (CARs) on the cell surface. The engineered T cell CARs recognize and kill the targeted tumor cells [152]. The CRISPR–Cas targeted gene delivery in the alpha locus of T cell CAR specific for CD19 led to stable CAR expression and increased T cell potency compared to conventional CAR T cells in a mouse model of acute lymphoblastic leukemia [153]. Multiplex targeting of the endogenous T cell receptor, β_2_ microglobulin (B2M), and programmed cell death protein 1 (PD-1) by CRISPR–Cas simultaneously resulted in gene-edited CAR T cells and potential antitumor activities [153,154].

(iv) Inhibition of checkpoint molecules: CRISPR–Cas inhibition of checkpoint molecules such PD-1 [154], lymphocyte activation gene 3 (LAG3) [155,156], and cytotoxic T-lymphocyte protein (CTLA4) [156] also demonstrated antitumor activities.

#### 3.2.4. Prospects in Therapeutics and Management of Infectious Diseases

The research data discussed above and the many more generated elsewhere provide greater insight into exploring new human diseases. CRISPR-edited constructs for the treatment of various genetic diseases such as sickle cell anemia, eye diseases, and cancer have shown promising effects in animal models. Although tissue-specific delivery of genome editors remains a key challenge in clinical applications of CRISPR, *in vivo* delivery tools are needed for cardiomyocyte, neurocytes, and immune cells for precise genome editing of genetic diseases and for analyzing genome-edited data. Human lung diseases, *viz*., cystic fibrosis causing a mutation in the cystic fibrosis transmembrane regulator (CFTR), was modified precisely by CRISPR-edited corrective CFTR sequences [157]. The use of CRISPR–Cas gene-targeted editing successfully removed Duchenne muscular dystrophy causing mutations in dystrophin proteins [158,159]. Patients with sickle cell disease and transfusion-dependent β-thalassemia were recently administered with BCL11A transcription factor targeting CRISPR–Cas9-edited CD34+ cell-enriched CTX001 and showed reactivation and a significant increase in fetal hemoglobin production [160]. Similar potential therapeutic strategies utilizing precise CRISPR-edited gene constructs are expected to be developed in the near future to correct the gene mutations of patients suffering from other human genetic disorders.

Several animal models such as chicken, cow, goats, and pigs are constructed by CRISPR to form bio-models to study humans and animal livestock. The ever-increasing demands of organs will be also reduced by these bio-models [161]. Specific human organs can be generated from animal blastocysts by disabling organ development in the host utilizing CRISPR [162]. In addition, longtime graft rejections will be improved by CRISPR. The applications of CRISPR–Cas9 in diverse fields such as transplantation can be performed using the manipulation of T cells and hematopoietic stem cells (HSCs) performed to cure blood cell-related diseases [163]. Transgenic pigs can be created with the removal of complications associated with porcine xenotransplantation [164]. Moreover, the CRISPR–Cas System is a potential tool in antiaging studies [165]. CRISPR–Cas system application for therapy is summarized in Table 6.

Brian Madeux, an American man with Hunter’s syndrome, received AAV-facilitated ZFN treatment in 2017. This highlighted the potential of gene editing for the first time in the treatment of genetic diseases. In cancer, CAR T cell therapy has been recognized as a “breakthrough therapy” and was approved in 2017 by the US Food and Drug Administration (FDA) for the treatment of leukemia and lymphoma [186], and soon, human trials of CRISPR will start. CRISPR technology not only helps in treatment but also in diagnosing diseases and reducing stress. CRISPR diagnostic kits and chips, which will be available to detect major diseases in no time, are upcoming [136]. Gene editing technologies facilitate the eradication of human diseases, for example, gene drive transfers and stabilizing altered genes in a wild population by a hundred percent. Similar to a study conducted in 2018, CRISPR crashed an entire population of mosquitoes by passing on infertility for a dozen generations using gene drive [187]. However, we cannot overlook the deleterious effects of gene drives on the human population from ethical perspectives.

### 3.3. In the Food Industry

Genome engineering of microorganism (bacteria and fungi) using CRISPR–Cas technology has been conducted in various research for improving cellular metabolism and production of valuable cellular metabolites [188]. Metabolic pathway such as the mevalonate pathway leading to the production of cholesterol and monoterpenes, which are commercially important in different traditional and modern pharmaceutical and cosmetic industries. Genome editing in yeast increases the mevalonate by more than 41-fold in comparison to non-edited strains [189]. The CRISPR–Cas system was also used to integrate the β-carotene synthetic pathway into the genome of *Escherichia coli* and for the modification of central metabolic and methylerythritol-phosphate (MEP) pathways for β-carotene overproduction [190]. The knockout of genes in *Corynebacterium* and the integration of synthetic single-stranded oligo-deoxyribonucleotides with the help of recombinase RecT produced high titers of γ-aminobutyric acid within a few weeks [191]. The production of succinate [192], galactaric acid [193], citric acid [194], and fatty acids have been enhanced by knocking out several genes and by optimizing the metabolic pathways [195]. Additionally, folic acid, biolipids, and nucleosides production from filamentous fungus *Ashbya gossypii* are enhanced by CRISPR–Cas9 markers with fewer nucleotide deletions and insertions [196].

#### Prospects in the Food Industry

CRISPR–Cas genome editing to increase the production of metabolites not only meets the increasing demand of society but also reduces pressure on the production sector and natural resources. CRISPR not only edits new organisms for metabolite production but also removes harmful contaminants formed during the process of formation. Mycotoxin contamination removal during the production of Monascus red, a food colorant, is one recent example [197]. CRISPR technology has shown high responses in crop improvement but has yet is to be explored in the field of plant synthetic biology and crop domestication.

Plant synthetic biology involves engineering traditional crops to design and produce novel bioproducts. CRISPR with its extraordinary feature of editing several genes together, i.e., multiplexing and integrating two or more genes at the predetermined location, i.e., gene stacking, is perfect for this. For example, to increase photosynthesis in C4 plants, the catalyzing properties of ribulose-1,5-bisphosphate carboxylase/oxygenase (Rubisco), an enzyme that catalyzes a rate-limiting step of photosynthesis, is increased by enhancing Rubisco production in plants. Therefore, three genes encoding Rubisco—larger subunit (LS), smaller subunit (SS) and Rubisco assembly factor 1 (RAF1)—were CRISPR-edited and overexpressed [112]. Several metabolic enzymes involved in crop yield, quality, and resistance if mutated in a frame by saturation mutagenesis would improve crops faster and better [198]. The application of the CRISPR–Cas system in the food industry is summarized in Table 7.

The domestication of nutritionally rich but wild crops such as sweet potato, banana, cassava, and quinoa has the potential to fight nutritional insecurity and food scarcity [60,61,72]. CRISPR technology is precise, cheap, and effective for inserting the desired gene for longer shelf-life and removal of unwanted genes from these crops. In CRISPR-edited crops, the CRISPR–Cas gene-editing tool possesses unique advantages over older endonucleases in genome editing tools. Before CRISPR, molecular scissors such as Zinc finger nucleases (ZFN), transcription activator-like effector nucleases (TALENs), and meganucleases (MN) were widely preferred for gene editing in crop plants. With the discovery of ZFN in 1986 [242] and of TALEN in 2011 [243], first-generation gene-editing tools and genome manipulations took heights. However, with the discovery of the prokaryotic adaptive immune system, there was the advent of a second-generation editing tool that was easy to construct [244], robust [245,246], feasible [247], and target-specific and was the most powerful technique for genome editing in plants [38]. According to the U.S. Department of Agriculture (USDA), CRISPR-edited foods are not the same as genetic modified organisms (GMOs) [88]. Therefore, such foods will be immune to regulations for GMOs.

**Figure 7:**
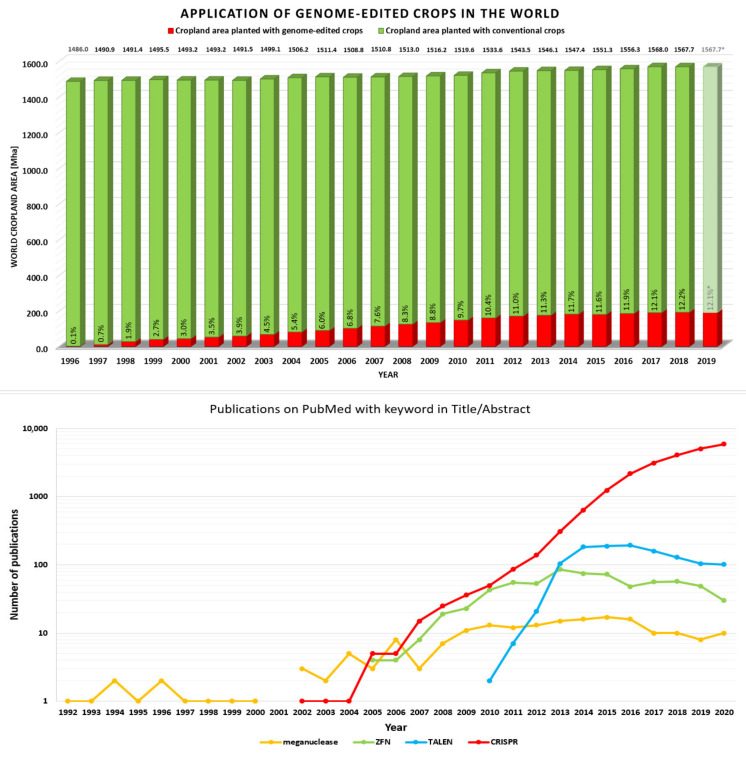
**Top:** Diagram represents the ratio of world cropland area planted with genome-edited crops (red column) and conventional crops (green column) since 1996. The numbers above the green columns represent a total world cropland area (sum of red and green column) in million hectares (Mha) in the corresponding year. The numbers above the red columns represent the percentage of world cropland area planted with genome-edited crops in the corresponding year. Due to absence of data for 2019 (total world cropland area), numeral values (colored gray and marked with an asterisk) are calculated according to the data of the total world cropland area in 2018. **Bottom:** Number of publications on PubMed with the keywords in their title/abstract. The following keywords were used: “meganuclease”, “ZFN”, “TALEN”, and “CRISPR” [250,251,252].

Global growing population and climate change will play a crucial role in the future of agriculture. The predicted human population in 2050 will reach 9.6 billion. This will result in a 60% higher demand for staple crops. In association with limited cropland areas and unpredictable weather conditions, food scarcity could become one of the biggest global issues in the future [248]. Genome editing appears to be a suitable technology to overcome this threat. The application of genome-edited crops in agriculture grew worldwide over the past decades (Figure 7). In 2018, genome-edited crops were planted on 12% of the world cropland area. Despite limited data about the global application of specific genome editing techniques in agriculture, high-effective systems are preferred. Site-specific nucleases such as TALENs and CRISPR–Cas systems have revolutionized biological research and have become widespread genome editing tool in crop plants [249]. For a better understanding of the CRISPR significance, several journal publications for each genome editing technique reported on PubMed is shown in Figure 7. The interest of the scientific community in novel techniques reflects their popularity and possibility for future applications. As seen in Figure 7, the popularity of ZFN slightly increased after 2005 and that in TALENs, in contrast, increased rapidly after the year 2010. However, CRISPR’s popularity is unambiguously higher in comparison to other genome editing techniques. Moreover, a slight decreasing number of publications related to ZFNs and TALENs in recent years is probably caused by CRISPR’s significance. Therefore, we expect further expansion of the CRISPR–Cas system application in the field of genome engineering.

## 4. CRISPR–Cas9: Ifs and Buts

A major limitation is the production of off-target effects in host cells, especially in mice embryos and adult human cells. The basic reason behind the off-target effects is the nonrecognition of target sequences by the 18–20 long nucleotide protospacer sequence in the sgRNA [253]. The sgRNA along with Cas9, despite widely used in genome editing, are limited by off-target effects and chromosomal translocation due to off-target cleavage. Choosing specific sequences on the genome and optimizing the sgRNA and Cas9 can reduce the RNA-guided endonuclease off-target mutations. Cas13a is more efficient in recognizing the target region than Cas9 because it can recognize PFSs at the 3′ end with 3′A, 3′U, or 3′C and the stem-loop structure made with 28 nt direct repeats. However, this problem still lingers and is complex when editing complex genomes such as that for a human. A higher proportion of off-target mutations occur in humans than in lower animals. The delivery of sgRNA and Cas9 to the host cell stands as a constant challenge to the scientific community. Scientists have used plasmids, viruses, and ribonucleoproteins for delivery purposes, but the process also suffers from limitations [253].

AquAdvantage salmon developed by AquaBounty Technologies is the first genetically engineered animal approved for consumption by the USA in 2015 and by Canada in 2016. AquAdvantage salmon reached a market size in half-reduced time compared to wild fish [254]. Due to its universality, CRISPR will probability also be applied in animals to increase muscle mass, to reduce diseases, to improve vitality, etc. However, much more dangerous is the application of CRISPR to eradicate diseases by eradicating disease vectors and invasive species. One of the examples is *Aedes aegypti*, a mosquito that transmits dengue fever. Researchers are developing genetically edited male-sterile mosquitos to prevent reproduction, with an aim to reduce the spread of disease. However, these ambitions could result in the extinction of entire species, with unpredictable environmental consequences [255].

Another controversial topic associated with CRISPR-mediated modifications in plants and animals is gene drive technology. The first success in gene drive technology was accomplished in 2011, when a gene inserted into the mosquito genome spread through the population, reaching more than 85% of the insects’ descendants. Gene drive is a genetic modification that is designed to spread through a population at higher-than-normal rates of inheritance. This CRISPR-based method alerts or silences a specific gene or inserts a new one. Gene drive actively copies a CRISPR-mediated mutation on one chromosome to its partner chromosome. This ensures that all offspring and the next generations will inherit the edited genome. Therefore, the application of gene drive in the environment possesses a much higher risk compared to genetically edited organisms, which has a 50% chance of inheriting the edited genes. Since 2014, scientists have engineered CRISPR-mediated gene drive systems in mosquitos, fruit flies, and fungi and are currently developing them in mice. No engineered gene drive organism has been released yet into the wild [255,256].

One of the most discussed topics associated with CRISPR is human genome editing. The possibility of modifying human DNA is subject to intense debates in ethics and law. There are three main discussed problems: (i) risk and uncertainty of the technology and its application, (ii) the human germline interference and responsibility towards future generations, and (iii) the legitimization of human genome editing measures with regard to concepts of therapy and enhancement. Despite the mentioned problems also being associated with other genome editing techniques, the introduction of the CRISPR–Cas system has accelerated an effort to find adequate solutions. The assumption of risk associated with editing the human genome prompted many national regulators to restrict or ban their human applications, e.g., any artificial modification of germline cells is prohibited in Germany; similarly, the National Institutes of Health (NIH) in the USA decided not to fund any use of gene-editing technologies in human embryos. The criticism of human genome editing opponents is also increasing with the glory of CRISPR–Cas. Many of them argue that scientists are “playing God”. Even the economic aspect is a bone of contention. Skeptics are afraid that CRISPR–Cas will become a beneficial technology only for rich people. In this unalluring scenario, a future society is divided into two main groups: the rich and healthy, and the poor and sick. Even if the uncertainty and risk associated with human genome editing could be minimized to an acceptable level, there are still several questions about whether it is ethically and legally justified to transfer these genetic modifications to future generations [257,258,259].

In therapeutics, CRISPR–Cas9 tools should be precisely and safely designed for long-term use. Human manipulation of genes that are passed on to organism via gene drive is risky and uncertain. The changes in the genome might lead to unknown changes undetected by any technology and may become a part of the human genome. Ethical concerns surround the use of CRISPR–Cas9 in humans and changing the descendant’s genome in any way [187]. Some studies have shown that CRISPR–Cas9 activates *TP53* overexpression through double-stranded breaks and leads to cell death [260]. Inactivation of *TP53* through CRISPR–Cas9 can decrease cell death but will increase off-targets and carcinogenicity. There are two separate papers published on 11 June 2018 linking increased risk of cancer cell development with gene editing [260,261]. Simultaneously, Chinese researchers worked on the editing *HBB* gene in nonviable embryos, producing mosaic embryo [262]. Among 86 embryos, approximately 82% of embryos survived and among which only 21 embryos were able to divide successfully but failed to show the desired genetic editing. In addition, the application of CRISPR–Cas9 in humans activates an immune response against Cas9, causing ill effects to the human body [114]. Researchers have modified the REC1 domain of Cas9, changing the epitope bound to T cell and thereby decreasing immune responses [263]. Recent reports on the advent of anti-CRISPR proteins project both opportunities and challenges in developing precise control over future CRISPR-mediated gene edits via the inhibition of Cas9 binding on edited DNA [264].

The most dreading limitation or fear associated with the CRISPR–Cas system is the development of biological warfare. Gene-editing can genetically engineer the bacteria and viruses to be used in biological attacks against humans or to cause widespread crop damage [265,266]. Potential applications of gene editing technology emerge from CRISPR science and technology, raising serious concerns on biosecurity as a “double-edged sword”. The potential nefarious biosecurity threats of CRISPR-mediated gene edits include the creation of novel neurotoxins/neuroweapons; virulence-enhanced *de novo* human and plant pathogens; increased human tolerance for soldiers against biological and chemical warfare; and bio-agents that cause human illness, degradation, disability, and lethality [267,268]. In comparison to the other genome/gene-editing technologies, CRISPR offers affordability, ease of use, and economical and extensive availability. Hence, the chances of misuse of these “weaponable entities” are also likely to be increased either by accident or by intentional and nefarious actors [267].

## 5. Conclusions and Future Perspectives

CRISPR–Cas is a sequence-specific nuclease able to edit the exact gene sequence, has revolutionized the field of biology, and has opened a new dimension in the field of genetic engineering and site-specific editing of nucleotide(s) within the malfunctioning gene. The tool’s efficiency, robustness, and vastness in editing a large number of genes found important functions and traits in plant breeding, livestock improvement, and biomedical engineering. The technology has wide application in editing bacterial, fungal, and yeast strains for modifying the pathways in secondary metabolites qualitatively and quantitatively. Auroramycin, a lactam ring-containing antibiotic was obtained recently from *Streptomyces roseosporus* by using a CRISPR–Cas9 gene cluster activation strategy [216]. Medicinal bioactive compounds such as morphine, thebaine, and new alkaloid compounds are also being produced in opium poppy (*Papaver somniferum* L.) using *Agrobacterium*-facilitated CRISPR–Cas via knockout genes [89].

**Figure 8:**
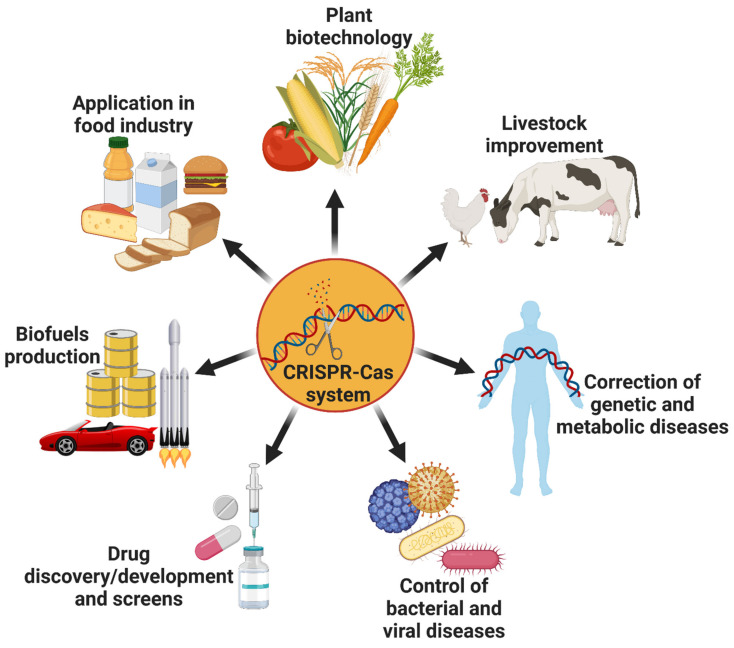
Applications of the CRISPR–Cas technology in various fields.

Serious climatic effects have increased dependency on cleaner fuel. Microbial production of biofuels decreases the dependency on natural resources and is an environment-friendly and economically efficient method of production. Advanced genome engineering methods such as CRISPR/Cas9 will optimize and improvise the bio-fuel production processes. Platform chemicals such as 3-methyl-1-butanol can be produced from renewable carbon source glucose directly by metabolically engineering industrially important *Pichia pastoris* [194]. Moreover, the use of CRISPR–Cas in hematologic diseases, infectious diseases, and malignant tumor via knockout, gene therapy, and gene editing has immense potential in therapeutics development (Figure 8).

In conclusion, the CRISPR–Cas system is a unique technology for gene editing. Studies summarized in this review represent only the first steps in the CRISPR–Cas era of genetic engineering. Indeed, the CRISPR–Cas system brings high-quality, desired benefits like never before. Fields of application for this technology also appear to be limitless. CRISPR–Cas, a highly precise genome editing tool, allows us to improve our quality of life. Our food will become more nutrient-dense without the presence of toxins or pathogens. The CRISPR-mediated improvement of quality and quantity, and resistance to viruses, herbicides, drought, salt, and cold have already been reported in several crops. However, the technology will bring a completely new generation of crops including novel varieties. The CRISPR revolution will affect the production of biofuels, new materials, and more. The CRISPR technology also bears the potential to revive extinct species in the future and even to create completely new species. However, misuse of CRISPR–Cas for gene editing could be a risk and danger; therefore, ethical discussion about CRISPR in the scientific community is important. Despite all risks, we believe that the application of CRISPR is a great opportunity for humanity and that exact gene editing will bring us a bright future. An age of CRISPR has already started.

## Figures and Tables

**Table 1:** Selected Cas proteins and their functions. Types (Roman numerals) are colored black, and subtypes (Roman numerals with a letter) are colored gray [14,21].

Protein	Association in Type or Subtype	Function
Cas1	I, II, IV, IV (assumed) III-A, III-B	DNA nuclease
Cas2	I, II, V III-A, III-B, VI (some)	RNA nuclease
Cas3	I	DNA nuclease and helicase
Cas4	II, V I (most)	DNA nuclease
Cas5	IV I-C, III (some)	pre-crRNA processing
Cas6	I (most), III-A, III-B	pre-crRNA processing
Cas7	I, III, IV	RNA recognition, crRNA binding
Cas8	I (most)	large subunit of Cascade complex
Cas9	II	DNA nuclease
Cas10	I (some), III (most)	large subunit of Csm or Cmr complex
Cas11	III I (some), IV (some)	small subunit of effector complexes
Cas12	V	crRNA processing, DNA nuclease
Cas13	VI	crRNA processing, RNA nuclease

**Table 2:** Cas protein association with CRISPR functional steps in type I–VI systems. An asterisk represents that protein being potentially fused to a large subunit in some subtypes. Underlined proteins are present in multiple copies. Proteins colored in gray are dispensable or missing in some subtypes or variants. Abbreviations: LS, large subunit; RT, reverse transcriptase; SS, small subunit; ?, unknown [14].

		Adaptation	Expression	Interference	
		Spacer Integration	pre-crRNA Processing	Effector Complex	Target Cleavage
class 1	type I	Cas1, Cas2, Cas4	Cas6	Cas7, Cas5, SS *, Cas8/LS	Cas3″, Cas3′
	type III	Cas1, Cas2, RT	Cas6	Cas7, Cas5, SS, Cas10/LS	Cas10/LS
	type IV	Cas1, Cas2	Cas6	Cas7, Cas5, SS, Csf1/LS	?
class 2	type II	Cas1, Cas2, Cas4	RNase III	Cas9	Cas9
	type V	Cas1, Cas2, Cas4	Cas12	Cas12	Cas12
	type VI	Cas1, Cas2	Cas13	Cas13	Cas13

**Table 3:** Classification of class 1 CRISPR–Cas systems [14].

Class	Type	Subtype	Variant	Native Target	Origin
1	I	I-A		DNA	*Archaeoglobus fulgidus* (AF1859, AF1870–AF1879)
		I-B		DNA	*Clostridium kluyveri* (CKL_2758–CKL_2751)
		I-C		DNA	*Bacillus halodurans* (BH0336–BH0342)
		I-D		DNA	*Cyanothece sp. 8802* (Cyan8802_0527–Cyan8802_0520)
		I-E		DNA	*Escherichia coli K12* (ygcB–ygbF)
		I-F	I-F1	DNA	*Yersinia pseudo-tuberculosis* (YPK_1644–YPK-1649)
			I-F2	DNA	*Shewanella putrefaciens CN-32* (Sputcn32_1819–Sputcn32_1823)
			I-F3		*Vibrio crassostreae J5 20* (VCR20J5_310088–VCR20J5_310108)
		I-G		DNA	*Geobacter sulfurreducens* (GSU0051–GSU0054, GSU0057–GSU0058)
	III	III-A		DNA + RNA	*Staphylococcus epidermidis* (SERP2463–SERP2455)
		III-B		DNA + RNA	*Pyrococcus furiosus* (PF1131–PF1124)
		III-C		DNA + RNA	*Methanothermobacter thermautotrophicus* (MTH328–MTH323)
		III-D		RNA?	*Synechocystis sp. 6803* (sll7067–sll7063)
		III-E		RNA?	*Candidatus Scalidua brodae* (SCABRO_02601, SCABRO_02597, SCABRO_02593, SCABRO_02595)
		III-F		DNA?	*Thermotoga lettingae TMO* (Tlet_0097–Tlet_0100)
	IV	IV-A			*Thioalkalivibrio sp. K90mix* (TK90_2699–TK90_2703)
		IV-B			*Rhodococcus jostii RHA1* (RHA1_ro10069–RHA_ro10072)
		IV-C		DNA?	*Thermoflexia bacterium* (D6793_05715–D6793_05700)

**Table 4:** Classification of class 2 CRISPR–Cas systems. An asterisk represents a variant that was formerly classified as variant I-U3 [14].

Class	Type	Subtype	Variant	Native Target	Origin
2	II	II-A		DNA	*Streptococcus thermophilus* (str0657–str0660)
		II-B		DNA	*Legionella pneumophila str. Paris* (lpp0160–lpp0163)
		II-C	II-C1	DNA	*Neisseria lactamica 020-06* (NLA_17660–NLA_17680)
			II-C2	DNA	*Micrarchaeum acidiphilum ARMAN-1* (BK997_03320–BK997_03335)
	V	V-A		DNA	*Francisella* cf. *Novicida Fx1* (FNFX1_1431–FNFX1_1428)
		V-B	V-B1	DNA	*Alicyclobacillus acidoterrestris* (N007_06525–N007_06535)
			V-B2	DNA	*Planctomycetes bacterium RGB_13_46_10* (A2167_01675–A2167_01685)
		V-C		DNA	*Oleiphilus* sp. (A3715_16885–A3715_16890)
		V-D		DNA	*Bacterium CG09_39_24* (BK003_02070–BK003_02075)
		V-E		DNA	*Deltaproteobacteria bacterium* (A2Z89_08250–A2Z89_08265)
		V-F	V-F1	DNA	*Uncultured archaeon* (NDOCEIEL_00008–NDOCEIEL_00011)
			V-F1*	DNA	*Bacillus thuringiensis HD-771* (BTG_31928)
			V-F2	DNA	*Uncultured archaeon* (ICDLJNLD_00049–ICDLJNLD_00052)
			V-F3		*Candidatus Micrarchaeota archaeon* (COU37_03050–COU37_03065)
			V-U1		*Gordonia otitidis* (GOOTI_RS19525)
			V-U2		*Cyanothece sp. PCC 8801* (PCC8801_4127)
			V-U4		*Rothia dentrocariosa M567* (HMPREF0734_01291)
		V-G		RNA	*Hot springs metagenome* FLYL01000025.1 (182949–185252)
		V-H			*Hypersaline lake sediment metagenome (JGI)* (Ga0180438_100006283)
		V-I		DNA	*Freshwater metagenome (JGI)* (Ga0208225_100001036)
		V-K			*Cyanothece sp. PCC 8801* (PCC8801_2993–PCC8801_2997)
	VI	VI-A		RNA	*Leptotrichia shahii* (B031_RS0110445)
		VI-B	VI-B1	RNA	*Prevotella buccae* (HMPREF6485_RS00335–HMPREF6483_RS00340)
			VI-B2	RNA	*Bergeyella zoohelcum* (HMPREF9699_02005–HMPREF9699_02006)
		VI-C		RNA?	*Fusobacterium perfoetens* (T364_RS0105110)
		VI-D		RNA	*Ruminococcus bicirculans* (RBI_RS12820)

**Table 5:** The application of CRISPR–Cas in plant biotechnology. Abbreviations: CBSD, Cassava brown streak disease; PUFA, polyunsaturated fatty acids.

Crops	Target Genes	Editing Process	Results	References
Biotic Factors				
Apple	*DIPM1*, *DIPM2*, *DIPM4*	Gene knockout	Resistance to fire blight disease	[58]
*Arabidopsis thaliana*	Non-coding/coding region of viral genome, *eIF(iso)4E*	Gene knockout	Virus resistance	[59]
Banana	*banana streak virus* genes	Gene inactivation	Virus resistant	[60]
Cassava	*eIF4E* isoforms *nCBP-1*, *nCBP-2*	Gene knockout	Partial resistance to CBSD	[61]
Cassava	*EPSPS*	Gene insertion and replacement	Herbicide resistant	[62]
Citrus	*PthA4*, *CsLOB1*	Gene knockout	Resistance to citrus canker	[63]
Citrus	*CsLOB1*	Gene knockout	Resistance to citrus canker	[64]
Cocoa	*TcNPR3*	Gene knockout	Increased resistance to *Phytophthora tropicalis*	[65]
Cotton	*clcud*	Gene disruption	Leaf curl disease resistant	[66]
Cotton	*Gh14-3-3d*	Gene knockout	Resistance to *Verticillium dahlia*	[67]
Cucumber	*eIF4E*	Gene knockout	Broad virus resistant	[68]
Flax	*EPSPS*	Gene insertion and replacement	Herbicide resistant	[69]
Grape vine	*VvWRKY52*	Gene knockout	Resistance to *Botrytis cinerea*	[70]
Grape vine	*MLO7*	Gene knockout	Resistance to powdery mildew	[58]
Potato	*ALS*	Gene insertion and replacement	Herbicide resistant	[71]
Potato	*Coilin*	Gene knockout	Increased resistance to potato virus Y	[72]
Rice	*OsERF922*, *OsSEC3A*, *OsSWEET13*	Gene mutations	Resistant to blast and bacterial blight	[73]
Rice	*CYP71A1*	Gene knockout	Broad-spectrum resistance to insect pests	[40]
Rice	*eIF4G*	Gene mutation	Resistance to *rice tungro spherical virus*	[74]
Rice	*ALS*	Gene insertion and replacement	Herbicide resistant	[42]
Rice	*EPSPS*	Gene insertion and replacement	Herbicide resistant	[75]
Rice	*C287T*, *ALS*	Multiplex genome editing	Herbicide resistance	[55]
Rice	*UVb1-1*	Multiplex genome editing	Resistance against false smut	[76]
Rice	*ALS*	Base editing	Herbicide resistant	[55]
Soybean	*ALS*	Gene insertion and replacement	Herbicide resistant	[77]
Tobacco	*AGO2*	Gene knockout	Virus resistance	[78]
Tomato	*SlMLO1*	Gene deletion	Powdery mildew resistant	[79]
Tomato	*SlJAZ2*	Gene truncation	Bacterial speck resistant	[80]
Watermelon	*ALS*	Base editing	Herbicide resistant	[57]
Wheat	*EDR1*	Gene knockout	Resistant to powdery mildew	[81]
Wheat	*TaMLO-A1*, *TaMLO-B1*, *TaMLO-D1*	Gene knockout	Resistant to powdery mildew	[82]
Abiotic Factors				
*Arabidopsis thaliana*	*UGT79B2 and UGT79B3*	Gene knockout	Cold, salt, and drought resistance	[83]
Barley	*ENGase*, *HvPM19*, *BolC.GA4.a*	Gene knockout	Grain number increase and dormancy control	[84]
False flax *(Camelina sativa)*	*FAD2*, *CsDGAT1*, *CsPDAT1*	Multiplex genome editing using CRISPR	Increased oleic acid and decreased PUFA	[34,36]
Grape vine	*IdnDH*	Gene insertion and replacement	Failure of tartaric acid biosynthesis or accumulation	[85]
Lotus	*SNF*, *SYMRK*, *LjLb1*, *LjLb2*	Gene knockout	Increased nitrogen accumulation	[86]
Maize	*Wx1*	Gene knockout	Increased amylose	[52]
Maize	*TMS5*	Gene knockout	Male sterile lines	[52]
Maize	*ARGOS8*	Gene insertion and replacement	Drought tolerance	[52]
Maize	*Dek42*	Gene knockout	Regulation of kernel development	[87]
Mushroom	*PPO*	Gene knockout	Browning-resistant mushroom	[88]
Opium poppy	*4′OMT2*	Gene knockout	Biomolecule synthesis	[89]
Orchids	*C3H*, *C4H*, *4CL*, *CCR*, and *IRX*	Gene knockout	Increased lignocellulose biosynthesis	[90]
Orchids	*PDS*	Gene insertion and replacement	Increasing quality and quantity	[91]
Potato	*StGBSS*	Gene knockout	Amylose synthesis	[92]
Potato	*StMYB44*	Gene knockout	Phosphate stress response	[93]
Potato	*ALS*	Gene knockout	Chlorsulfuron resistance	[94]
Rice	*Gn1a*, *DEP1*, *GS3*	Gene knockout	Enhanced grain size and number and dense erect panicles	[95]
Rice	*OsGn1a*	Gene knockout	Grain number	[30,31]
Rice	*OsGS3*	Gene knockout	Grain size	[30,31]
Rice	*TaGW2*, *OsGW5*, *OsGLW2*, or *TaGASR7*	Gene knockout	Grain weight	[30,31]
Rice	*OsDEP1*, *TaDEP1*	Gene knockout	Panicle size	[30,31]
Rice	*LAZY1*	Gene knockout	Tiller spreading	[30,31]
Rice	*OsAAP3*	Gene knockout	Increased tiller number	[30,31]
Rice	*GW2*, *GW5*, and *TGW6*	Gene knockout	Gain weight improvement	[96]
Rice	*OsPDS*, *OsMPK2*, *OsBADH2*	Gene knockout, Indels	Edited abiotic stress tolerance	[38]
Rice	*OsDERF1*, *OsPMS3*, *OsEPSPS*, *OsMSH1*, *OsMYB5*	Gene knockout	Edited drought tolerance	[97]
Rice	*OsHAK-1*	Gene knockout	Strongly reduced content of ^137^Cs^+^	[98]
Rice	*OsPRX2*	Gene knockout	Serious defects in leaves, stomal opening under K^+^-deficient conditions	[99]
Rice	*OsAnn3*	Gene knockout	Decreased cold tolerance	[100]
Rice	*SBEIIb*	Gene knockout	Higher amylose content	[37]
Rice	*TMS5*	Gene knockout	Thermosensitive male sterile lines	[51]
Rice	*csa*	Gene knockout	Photosensitive male sterile lines	[95]
Rice	*OsMATL*	Gene knockout	Haploid seed formation	[54]
Rice	*ACCase gene*	Base editing	Haloxyfop-R-methyl resistant	[101]
Rubber	*TK 1-FFT*	Gene knockout	Rubber biosynthesis	[102]
Soybean	*GmSPL9a*, *b*, *c*	Gene insertion and replacement	Improved yield	[103]
Tomato	*SlAGL6*	Gene knockout	Parthenocarpic fruit production under heat stress conditions	[39]
Tomato	*SlIAA9*	Gene knockout	Production of parthenocarpic plants	[104]
Tomato	*RIN*	Gene knockout	Improved shelf life	[105]
Tomato	*AP2a*, *NOR*, *TDR4*, *MBP7*	Gene knockout	Delayed fruit ripening	[106]
Tomato	*SlAGO7*	Gene knockout	Increased growth	[107]
Tomato	*SlNPR1*	Gene knockout	Role of *SINPR1* in drought resistance	[108]
Wheat	*GW2*	Base editing	Increased grain and protein content	[32]
Wheat	α-gliadin family members	Indels	Decreased gluten	[35]
Wheat	*PinB*	Gene insertion and replacement	Genetic improvement	[109]
Wheat	*TaWaxy*, *TaMTL*	Gene insertion and replacement	Induction of haploid plants	[110]
Wheat	*Ms45*	Base editing	Male-sterility	[53]

**Table 6:** The application of CRISPR–Cas in therapeutics. Abbreviations: HBV, hepatitis B virus; IE protein, immediate early protein; RBC, red blood cells; ROS, reactive oxygen species.

Disease	Target Genes	Editing Process	Results	Reference
Cataracts	*EGFP*, *Crygc*	Indels	Gene correction of *Crygc* gene	[166]
Chronic granulomatous disease (CGD)	*CYBB*	Single point mutation	Restoration of ROS activity on phagocytic cells	[167]
Coronary heart disease	*PCSK9*	Insertion/deletion	Prevent coronary heart disease	[168]
Cystic fibrosis (CF)	*CFTR*	Base editing	Normal recovery of CFTR expression	[157]
Diabetes mellitus type 1 (DM1)	*DMPK*	Gene editing	Play important role in disease etiology	[169]
Duchenne muscular dystrophy (DMD)	exon44	Gene deletion	Restoration of dystrophin protein	[116]
Duchenne muscular dystrophy (DMD)	*Dmd*	Gene deletion	Restoration of dystrophin protein	[170]
Hemophilia B andA	*F9* and *F8*	Gene knock-in and alteration	Control bleeding process	[118]
Hearing loss	*Tmc1*	Gene disruption	Prevention of deafness	[120]
Hematopoietic diseases	*BCL11A*	Gene deletion	Increased production of γ-hemoglobin	[119]
Huntington disease (HD)	*HTT*	Small targeted deletions	Terminating HTT expression	[171]
Sickle cell disease (SCD)	*HBB*	Indels	RBC formation	[121]
Sickle cell disease (SCD)	*BCL11A*	Gene interference	RBC formation	[172]
WPW syndrome	*PRKAG2*	Insertion/deletion	Correction of PRKAG2 cardiac syndrome	[173]
α1-antitrypsin	*hSERPINA1*	Gene disruption	Reduced expression of liver fibrosis markers	[117]
β-thalassemia	*HBB*	Mutation deletion	RBC formation	[174]
Cancer Tissue Therapy				
Ataxia-telangiectasia	*ATM*	Gene knockout	Increased ATM-independent repair mechanism	[175]
Bladder cancer	*ATG5*	Gene knockout	Cytotoxicity suppression	[149]
Cervical cancer	*E6* and *E7*	Gene knockout	Anti-tumor activities	[176]
Cholangiocarcinoma	*BAP1*		Loss of malignancy	[151]
Colorectal cancer	*Acvr1b*, *Acvr2a*, and *Arid2*	Gene knockout	Suppression of cancer	[150]
Human lung adenocarcinoma A549 cells	*CD38*	Gene knockout	Inhibited anchorage-independent cell growth	[148]
Hypertrophic cardiomyopathy (HCM)	*MYBPC3*	Mutation correction	Maintain sarcomere structure and regulate relaxation/contraction	[177]
Leukemia and lymphoma	*TRAC* locus	Knock-in	Enhanced anti-tumor capability	[152]
Melanoma	*PD-1*, *LAG3,* and *CTLA4*		Anti-tumor activities	[155]
Nijmegen breakage syndrome	*NBS1*	Mutation correction	Decreased susceptibility of cancer	[178]
Non-small cell lung cancer	*NPM1*	Gene knockout	Anti-tumor activities	[179]
Tumor	*B2M*	Gene disruption	Anti-tumor activities	[153]
Tumor	*PD-1*	Gene disruption	Anti-tumor activities	[154]
Cell Therapies				
Lung and esophageal cancer	*PD-1* of T cells	Gene knockout	Clinical trials	[180]
Viral Infection				
Chimeric hepatitis B	Repeat regions	Gene inactivation	Eradication of HBV infection	[181]
HIV	*LTR U3 region*	Gene knockout	Loss of LTR expression	[126]
HIV	*CCR5* on T cells	Gene knockout	Reduced entry of virus	[127]
HIV	*CXCR4*	Gene knockout	Resistant to X4 HIV virus	[128]
HIV	*CXCR4* and *CCR5*	Gene knockout	Cytotoxicity of cells	[129,130]
HIV	Subgenomic particles	Gene knockout	Removal of proviral DNA	[131]
HSV-1	*ICP0*, *ICP4*, *ICP27*		Abrogation of viral infection	[137]
Human cytomegalovirus	*UL122/123*		Decreased IE protein expression, reduced production of new virions	[141]
Human polyomavirus (JCV)	N-terminal of T-antigens		Suppressed viral replication	[143]
Kaposi’s sarcoma-associated herpesvirus	*LANA*		Decreased episomal load	[140]
Bacterial Infection				
Burkitt lymphoma Hodgkin’s disease	*BART5*, *BART6*	Gene deletion	Suppressing the viral replication	[139]
*Escherichia coli*	*ftsA*, *asd*, *msbA*, *nusB*	Gene knockout	Antibiotic resistance	[182]
*Escherichia coli*	*NDM-1*, *CTX-M-15*	Gene truncation	Carbapenem resistance	[183]
*Escherichia coli*	*blaTEM*, *blaSHV*	Gene truncation	Re-sensitization to β-lactam resistance	[184]
*Staphylococcus aureus*	*aph-3*, *mecA*	Gene deletion	Confer resistance against kanamycin	[185]

**Table 7:** The application of CRISPR–Cas in the food industry. Abbreviations: CRISPRi, CRISPR interference; GFP, green fluorescent protein; MUCICAT, multicopy chromosomal integration using CRISPR-associated transposases.

Target Species	Target Gene(s)	Editing Process	Results	References
Bacteria				
*Ashbya gossypii*	*ADE2*	Nucleotide deletions	Production of folic acid, biolipids, and nucleoside	[196]
*Bacillus smithii*	*pyrF*, *amyE*, *trpC2*	Gene deletion and insertion/recombination	Capable of using five and six carbon containing sugars	[199,200]
*Bacillus subtilis*	*cypX*, *yvmA*	Gene deletion and insertion	Production of several enzymes and low molecular weight substances	[201]
*Clostridium autoethanogenum*	*adh*, *2*, *3-bdh*	Gene deletion and insertion	Production biofuels and 2,3-butanediol utilizing CO, CO_2_, and H_2_	[202]
*Clostridium beijerinckii*	*Ack*, *adhE*	Gene deletion and insertion	Biofuels and biochemicals production	[203]
*Clostridium cellulolyticum*	*pyrF*, *MspI*	Gene deletion and insertion	Model for production of renewable biochemicals	[204]
*Clostridium ljungdahlii*	*pta*, *adhE1*, *ctf* and *pyrE*	Gene deletion	Production of ethanol from synthesis gas	[205]
*Clostridium pasteurianum*	*cpaAIR*	Gene deletion	Potential biofuel from conversion of waste glycerol into ethanol	[206]
*Corynebacterium glutamicum*	*Pgi*, *pck*	CRISPRi	Increased production of γ-aminobutyric acid	[207]
*Cyanobacteria*	*glgC*, *gltA*, *ppc*	Gene deletion and insertion	Increased production of succinate	[208]
*Escherichia coli*	Growth-related genes	CRISPRi, multiplexed CRISPRi, multiplexed RNA, RNA targeting, MUCICAT	Enhanced model for production of bioproducts and enzymes	[209,210]
*Lactobacillus reuteri*	*lacL*	Gene deletion and insertion	Model for new single stranded DNA editing	[211]
*Streptococcus thermophiles*	Growth-related genes	Genome editing	Pro-biotic activity and fermentation	[212]
*Streptomyces albus*	*redF*	Gene deletion	Bioactive products	[213]
*Streptomyces coelicolor*	*actII-orf4*, *glnR*, *redF*	Gene deletion and insertion, CRISPRi	Improvement in genome editing efficiency as a model	[214]
*Streptomyces rimosus*	*zwf2*, *devB*	Gene mutation and disruption	Increased yield of oxytetracycline	[215]
*Streptomyces roseosporus*	PKS gene cluster	Gene cluster activation	Production of auroramycin and its aglycon	[216]
*Streptomyces viridochromogenes*	Biosynthetic gene cluster	Gene knock-in	Production of pentangular type II polyketide	[213]
*Tatumella citrea*	*tkrA*, *glk*	MUCICAT, allelic exchange	Successful application of the method, multiplex genome editing system	[217,218]
Yeast				
*Agaricus bisporus*	*PPO*	Gene disruption	Non-transgenic variety	[88]
*Alternaria alternata*	*pksA*, *brm2*	Gene deletion/insertion	Established pyr4 as selection marker and GFP for protein tagging	[219]
*Aspergillus aculeatus*	*alba*	Gene mutation	Potential genome editor in filamentous fungi	[220]
*Aspergillus brasiliensis*	*pyrG*	Gene mutation	Potential genome editor in filamentous fungi	[221]
*Aspergillus carbonarius*	*ayg1*	Gene mutation	Potential genome editor in filamentous fungi	[220,222]
*Aspergillus fumigatus*	*pksP*	Gene cleavage	Potential toolbox for decreasing pathogenicity	[223]
*Aspergillus luchuensis*	*brlA*, *niaD*, *amyA*	Gene mutation	Potential genome editor in filamentous fungi	[220,224]
*Aspergillus nidulans*	*yA*	Gene mutation	Potential genome editor in filamentous fungi	[220]
*Aspergillus niger*	*udh*	Gene deletion/insertion	Increased production of galactaric acid	[193]
*Aspergillus oryzae*	*Ku-70*, *ligD*	Gene deletion/insertion	Construct plasmid for targeted mutagenesis	[225]
*Candida albicans*	*ADE2*	Recombination and multiplexed recombination	Genetic manipulation for increased biproducts	[226]
*Candida glabrata*	a GPI-anchored aspartyl protease, a putative serine/threonine kinase	Gene cleavage	Understanding *C. glabrata* virulence *in vivo*	[227]
*Cryptococcus Neoformans*	*ADE2*, *L41*, *Tsp2*	Gene cleavage	Genome engineering in higher fungi	[228]
*Fusarium fujikuroi*	*tHMGR*, *Cps/Ks*	Gene knockout	Improved gibberellic acid production	[229]
*Ganoderma species*	*ura3*	Gene cleavage	Codon optimization	[230]
*Huntiella omanensis*	*MAT1-2-7 gene*	Gene inactivation	Role in sexual reproduction	[231]
*Kluyveromyces lactis*	*GAL80*, *YKU80*	Gene deletion and insertion	Enhanced production strain	[232]
*Myceliophthora thermophila*	*cre-1*, *res-1*, *gh1-1*, and *alp-1*	Multigene disruption	Enhanced hypercellulase production	[233]
*Neurospora crassa*	*CLR-2*, *GSY-1*	Gene replacement	Increased expression of cellulase and luciferase	[234]
*Penicillium chrysogenum*	*pks17*, *amdS*	Gene cleavage	Potential genome editor in filamentous fungi	[235]
*Phytophthora sojae*	*RXLR*, *Avr4/6*	Gene replacement	Control pathogenicity	[236]
*Pichia pastoris*	*AOX1*, *MPPI*	Gene deletion	Production of *iso*-pentanol	[194]
*Saccharomyces cerevisiae*	Growth-related genes	CRISPRa, CRISPRi, Multiplex metabolic engineering	Increased bioproducts	[189,232]
*Schizosaccharomyces pombe*	*rrk1*	Gene cleavage	CRISPR toolbox in *S. pombe*	[237]
*Talaromyces atroroseus*	*PKS*	Gene cleavage	Production of polyketide-nonribosomal peptide	[238]
*Trichoderma reesei*	*ura5*	Homologous recombination	Tool for genome engineering	[239]
*Ustilago maydis*	*bE1*, *bW2*	Gene disruption	Increased DNA repair system	[240]
*Yarrowia lipolytica*	*PEX10*, *KU70*, and *MFE1*	Gene disruption	Increased synthesis and storage of lipids	[241]
